# Antidepressants Trial in Parkinson's Disease (ADepT-PD): protocol for a randomised placebo-controlled trial on the effectiveness of escitalopram and nortriptyline on depressive symptoms in Parkinson’s disease

**DOI:** 10.1186/s12883-022-02988-5

**Published:** 2022-12-12

**Authors:** A Schrag, C Carroll, G Duncan, S Molloy, L Grover, R Hunter, R Brown, N Freemantle, J Whipps, M. A Serfaty, G Lewis

**Affiliations:** 1 Department of Clinical and Movement Neurosciences, UCL Queen Square Institute of Neurology, University College London, London, UK; 2 Department of Neurology, Royal Free London NHS Foundation Trust, London, UK; 3 Faculty of Medicine and Dentistry, University of Plymouth, Plymouth, UK; 4 NHS Lothian, Edinburgh, UK; 5 Department of Neurosciences, Imperial College Healthcare NHS Trust, London, UK; 6 Research Department of Primary Care and Population Health, University College London, London, UK; 7 Department of Psychology, Institute of Psychiatry, King’s College London, London, UK; 8 Comprehensive Clinical Trials Unit, University College London, London, UK; 9 PPI Representative, Plymouth, UK; 10 Division of Psychiatry, UCL, London, UK; 11 Priory Hospital North London, London, UK

**Keywords:** Parkinson’s disease, Depressive symptoms, Escitalopram, Nortriptyline, Randomised controlled trial, Clinical effectiveness, Cost-effectiveness

## Abstract

**Background:**

Depressive symptoms are common in patients with Parkinson’s disease and depression is a significant predictor of functional impairment, reduced quality of life and general well-being in Parkinson's disease. Despite the high prevalence of depression, evidence on the effectiveness and tolerability of antidepressants in this population is limited. The primary aim of this trial is to establish the clinical and cost effectiveness of escitalopram and nortriptyline for the treatment of depression in Parkinson’s disease.

**Methods:**

This is a multi-centre, double-blind, randomised placebo-controlled trial in 408 people with Parkinson’s disease with subsyndromal depression, major depressive disorder or persistent depressive disorder and a Beck Depression Inventory-II (BDI-II) score of 14 or above. Participants will be randomised into one of three groups, receiving either escitalopram, nortriptyline or placebo for 12 months. Trial participation is face-to-face, hybrid or remote. The primary outcome measure is the BDI-II score following 8 weeks of treatment. Secondary outcomes will be collected at baseline, 8, 26 and 52 weeks and following withdrawal, including severity of anxiety and depression scores as well as Parkinson’s disease motor severity, and ratings of non-motor symptoms, cognitive function, health-related quality of life, levodopa-equivalence dose, changes in medication, overall clinical effectiveness, capability, health and social care resource use, carer health-related quality of life, adverse effects and number of dropouts.

**Discussion:**

This trial aims to determine the effectiveness of escitalopram and nortriptyline for reducing depressive symptoms in Parkinson’s disease over 8 weeks, to provide information on the effect of these medications on anxiety and other non-motor symptoms in PD and on impact on patients and caregivers, and to examine their effect on change in motor severity.

**Trial registration:**

ClinicalTrials.gov Identifier: NCT03652870

Date of registration – 29^th^ August 2018

**Supplementary Information:**

The online version contains supplementary material available at 10.1186/s12883-022-02988-5.

## Background

Parkinson’s disease (PD) is a progressive neurological disorder that leads to increasing disability and functional decline. Approximately 35% of individuals with PD experience co-morbid depression or depressive symptoms throughout the course of their illness [1–3]. Depression is associated with functional impairment, cognitive decline, faster disease progression and reduced quality of life [4, 5]. Despite the high prevalence of depression, there is currently limited evidence on the efficacy of antidepressants for treating symptoms of depression in PD. The most common medications for treating depressive disorders in the UK are selective serotonin reuptake inhibitors (SSRIs), as recommended by the National Institute for Health and Care Excellence (NICE) guidelines [6]. However, SSRIs are often used cautiously in PD due to some preclinical studies and clinical reports of worsening parkinsonism [7, 8]. Additionally, other side effects such as fatigue or postural hypotension can occur and may already be pre-existing in PD [9, 10]. Furthermore, there have been reports about an increase in falls in patients on SSRIs, [11] and very rarely serotonin syndrome has been reported [12]. Tricyclic antidepressants (TCA), which have mixed properties including serotonin reuptake inhibition and noradrenaline reuptake inhibition, as well as anticholinergic and antihistamine actions, have similar efficacy to SSRIs [13, 14]. Their anticholinergic properties can also reduce both tremor and insomnia in PD. The TCA nortriptyline has also been suggested to have neuroprotective properties in pre-clinical studies by inhibition of aggregation and neurotoxicity of alpha-synuclein [15]. However, TCAs are currently recommended only as second line treatments for depression in PD due to increased risk of adverse side effects including, but not limited to, orthostatic hypotension, dry mouth, constipation, urinary retention, memory impairment, hallucinations and confusion. Some existing trial evidence supports the efficacy for both TCAs [13, 16, 17] and SSRIs [18] for depressive symptoms in PD but overall there is surprisingly little evidence available on the effectiveness of these antidepressants in PD, particularly in a real-life NHS setting in a representative population. Only two trials comparing SSRIs and TCAs have been reported, both with relatively small sample sizes of 15 to 18 per group respectively [16, 19], and of a treatment duration of 8 weeks and 30 days. Both SSRIs and TCAs, compared to placebo, reported improvement of depression, anxiety, dysphoria, vegetative symptoms and tolerability of the active agents. Whilst evidence suggests SSRIs are cost-effective compared with TCAs for major depression [14], no studies have explored this in depression in PD, and little information exists on the effect of antidepressant treatment in PD on health-related quality of life, carer burden and capability. No clinical trials have assessed the effect of nortriptyline on clinical disease progression in PD.

Due to the range of inconclusive and inconsistent evidence on both SSRIs and TCAs in the treatment of depression in PD, there is a need for conclusive trial evidence on both the clinical and cost-effectiveness of these two treatments to guide evidence-based treatment of depression in PD in the NHS in the short and longer term over 12 months, provide evidence on the effect on anxiety, cognition and other non-motor symptoms, health-related quality of life and carer burden, and to investigate potential effects on motor function over one year.

### Objectives

The primary objective of this trial is to establish the clinical and cost-effectiveness of escitalopram and of nortriptyline at 8 weeks compared to placebo in the treatment of depression in PD, in addition to standard psychological care in the NHS.

The secondary objectives are to establish i) whether after one year of treatment parkinsonism has deteriorated less in patients with Parkinson’s disease with depression on nortriptyline than on placebo; ii) the difference in adverse reactions between escitalopram and nortriptyline; iii) the long-term (after one year of treatment) clinical effectiveness and cost-effectiveness of escitalopram and nortriptyline compared to placebo; iv) the clinical effectiveness of escitalopram and of nortriptyline compared to placebo on anxiety, cognition, overall function, and health-related quality of life; and v) whether after one year of treatment parkinsonism has deteriorated more in patients with Parkinson’s disease with depression on escitalopram than on placebo.

## Methods/Design

### Study design

**Fig. 1:**
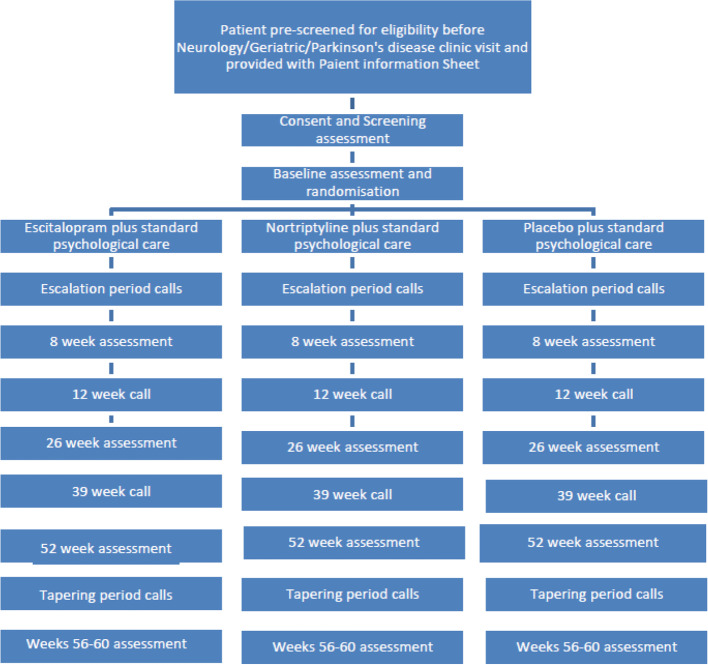
Trial diagram

ADepT-PD is a multi-centre, double-blind, phase III randomised controlled trial with an internal pilot study and a parallel arm design (see Fig. 1). Participants will be randomly allocated 1:1:1 to receive escitalopram or nortriptyline or placebo.

### Participants and setting

Specialist PD clinicians will identify participants from approximately 30 sites across the United Kingdom, including neurology, care of the elderly, PD clinics, mental health trusts, primary care and community settings. The trial will be publicised through clinical networks, charities primary care surgeries and social media. Potential participants will be contacted for a pre-screening call and sent a participant information sheet detailing the trial. Suitability will be assessed by a trained clinician according to the eligibility criteria and fully informed consent will be retrieved from each participant.

Participants will be included if they: 1) have a diagnosis of idiopathic PD, including at least two of the three cardinal signs: rigidity, bradykinesia and rest tremor, 2) are aged 18 or above, 3) fulfil operationally defined subsyndromal depression (presence of two or more depressive symptoms, at least one of which has to include depressed mood or anhedonia, or diagnostic (*DSM-V*) criteria for a depressive disorder (i.e., major depressive disorder or persistent depressive disorder), 4) have a BDI-II score of 14 or above, 5) have optimised or stable antiparkinsonian medication for at least 4 weeks prior to randomisation with no plan to change up to primary endpoint (8 weeks) and 6) provide fully informed consent.

The study exclusion criteria are: 1) pregnancy, breastfeeding or childbearing potential without effective contraception, 2) inability to understand the participant information sheet or questionnaires, 3) a Montreal Cognitive Assessment (MoCA) score below 16 or lack of capacity to consent, 4) treatment with an antidepressant within 4 weeks of enrolment (except for a small dose of amitriptyline up to 30mg for indications other than depression), 5) severe liver failure, 6) contraindications to the study medication, including known QT-interval prolongation or congenital long QT syndrome, recent myocardial infarction (< 3 months), any degree of heart block or other cardiac arrhythmias precluding treatment with nortriptyline or escitalopram according to clinical judgement, 7) medications contraindicated on nortriptyline or escitalopram, including a). non-selective and selective irreversible monoamine oxidase (MAO) inhibitors within 14 days (the antiparkinsonian selective reversible MAO-B inhibitors rasagiline, selegiline and safinamide are not contraindicated) and b). Concomitant QT prolonging drugs, including domperidone, apomorphine at high doses (single dose or hourly rate of > 6mg), certain neuroleptics (not quetiapine or clozapine), quinine, class IA and III antiarrhythmics (amiodarone, dronedarone and disopryamide), the antihistamines astemizole, mizolastine, the antimicrobial agents sparfloxacin, moxifloxacin, erythromycin IV, pentamidine, anti-malarian treatment), and some antiretrovirals), 8) suicidal ideation or intent on the BDI-II item 9 and who, after clinical review of risk using the standardised Suicide Risk Management Protocol, need to be referred for immediate treatment, 9) participation in another trial of an investigational medicinal product or device within the last 30 days of randomization, and 10) any clinical condition which in the opinion/ clinical judgement of the investigator would make the patient unsuitable for the trial due to safety concerns.

### Randomisation and blinding

Following consent, participants will be randomly assigned to receive either escitalopram, nortriptyline or placebo. Randomisation will be completed using a minimisation algorithm by the randomisation service (Sealed Envelope Ltd., London). Randomisation will be done using a minimisation algorithm provided by the randomisation service (Sealed Envelope.com). The factors minimised on will include site, depression severity (BDI-II 14–19/20–63), Hoehn & Yahr disease severity staging in the ON medication stage (≤ 2.0/ ≥ 2.5), amitriptyline usage (yes/no)), clonazepam/benzodiazepine usage (yes/no), gabapentin/pregabalin usage (yes/no) and pramipexole/dopamine antagonist usage (yes/no).

The trial statistician will generate unique kit codes for every active/placebo trial medication bottle which will be entered into the web-based, password-protected, secure randomisation service provided by the independent data management company.

To maintain study blinding, all trial medication will be provided in bottles of identically appearing tablets. Site staff completing assessments will be kept blind to trial arm allocation, as will be the participants and the trial team and analysts.

### Assessment procedures

Participants will undergo assessment at baseline and 8, 26, 52 and following withdrawal at 56–60 weeks. All assessments will be performed by trained and suitably qualified members of the clinical trial team and completed either face-to-face or using a remote video conferencing software.

Assessments will include quantification of depressive and anxiety symptoms, PD motor features and non-motor symptoms, cognitive function, health-related quality of life, levodopa-equivalence dose, changes in medication, overall clinical effectiveness, capability, health and social care resource use, carer burden, adverse side effects and number of dropouts.

### Intervention

Participants will receive either:

1. Escitalopram, target dose: 20mg if 65 years and under; or 10mg if > 65 years or in those with hepatic impairment
2. Nortriptyline, target dose: 100mg if 65 years and under, or 50mg if > 65 years or in those with hepatic impairment
3. Placebo

The initial dosage of trial medication will be as follows:

1. 5mg escitalopram increased by 5mg per day, at two-weekly intervals, to a maximum target of 20mg escitalopram per day
2. 25mg nortriptyline increased by 25mg per day, at two-weekly intervals, to a maximum target of 100mg nortriptyline per day

The dosing regime is shown in Tables 1 and 2. The dosage may decrease to the previous dose level if intolerable side effects occur.

**Table 1:**
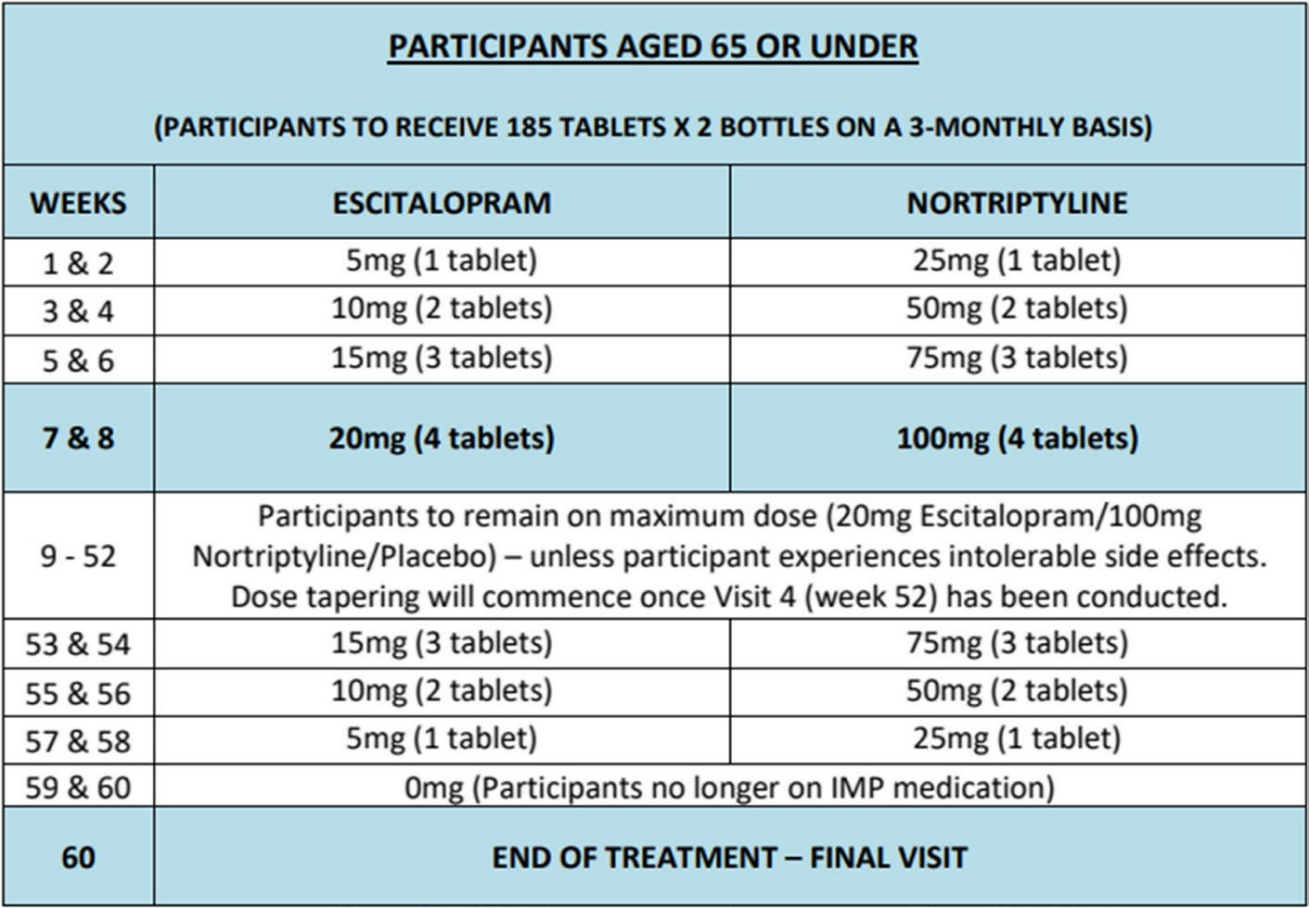
Dosing regime for participants aged 65 or under

**Table 2:**
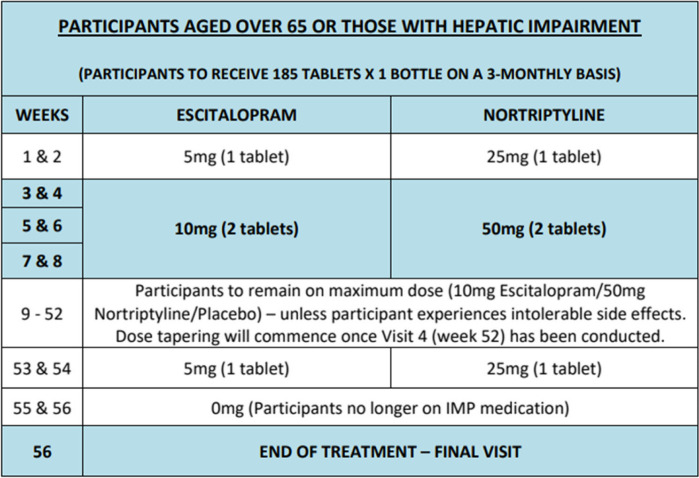
Dosing regime for participants aged over 65 or those with hepatic impairment

The medication will be sent to the participant’s home following randomisation. The total treatment duration will be 56 weeks.

Standard psychological care will be provided in conjunction, this may include referral to an Improving Access to Psychological Therapies service to receive Cognitive Behavioural Therapy or other forms of therapy.

After the primary endpoint at 8 weeks, all participants will continue on the same dose (*if applicable*) until the study visit at 52 weeks with an intermediate assessment at 26 weeks. If there is failure to respond to therapy and there is a clinical need to treat depression with an alternative agent, trial medication will be tapered and stopped and alternative therapy introduced as appropriate by their GP/clinician. If there is a need to stop the trial medication due to lack of efficacy or due to side effects, or the wish of the participant, participants will be encouraged to attend all study visits to obtain follow up assessments.

At the study assessment after 52 weeks on medication, the trial medication will be tapered off in dose reductions of 25mg for nortriptyline and 5mg for escitalopram every two weeks (8 weeks for participants 65 years or under and 4 weeks for participants > 65 years or those with hepatic impairment). Blinding of the participants and trial team will be maintained until the trial medication has been withdrawn but an unblinded member of the trial team will communicate the treatment allocation to the GP (and not the participant or the trial team) if they request unblinding earlier. For those who may wish to continue treatment at study end, liaison will be made with the GP to communicate the treatment allocation following unblinding by an unblinded member of the trial team.

### Adherence and retention

Participants will be required to record uptake and timing of trial medication using a dosing diary. A member of the research team will contact the participant at each time point as a reminder to complete the diary. A dosing diary discussion with the assessor will take place at baseline, weeks 8, 26 and 52.

If a participant chooses to discontinue their trial treatment, they will be encouraged to continue to be followed up as closely as possible to the follow-up schedule defined in the protocol. Participants who withdraw early from the trial will be asked to complete the BDI-II questionnaire to help evaluate their depressive symptoms at the time of withdrawal. Data already collected will be kept and included in analyses according to the intention to treat principle for all participants who stop follow up early.

### Primary outcome – Depressive symptoms

The primary outcome will be depressive symptoms after 8 weeks of treatment as measured using the Beck Depression Inventory-II (BDI-II), to assess effectiveness against placebo.

### Secondary outcomes

The following secondary outcomes will be measured at baseline, 8, 26 and 52 weeks of treatment and following withdrawal (Table 3 and supplementary table 1).

**Table 3:** Outcome measures

**Domain**	**Measure(s)**	**Timepoint (week)**
Motor and non-motor PD symptoms	Movement Disorder Society Unified Parkinson’s Disease Rating Scale (MDS-UPDRS) during “On”	0, 8, 26, 52 and 56–60
	MDS-UPDRS part III during “Off” (optional)	0, 8, 26, 52 and 56–60
	Wearable sensor (optional)	0, 26 and 56–60
Depression	Beck Depression Inventory (BDI-II)	0, 26, 52 and 56–60
	Patient Health Questionnaire 9 (PHQ-9)	0, 8, 26 and 52
Anxiety	Parkinson Anxiety Scale (PAS)	0, 8, 26 and 52
Cognitive function	Montreal Cognitive Assessment (MoCA)	0, 8, 26, 52 and 56–60
Health-related Quality of life	EQ-5D-5L	0, 8, 26 and 52
Capability	ICECAP-O	0, 8, 26 and 52
Overall clinical effectiveness	Clinical Global Impression (CGI)	0, 8, 26 and 52
Health and social care resource use	Modified Client Service Receipt Inventory (CSRI) which incorporates the modified iVICQ	0, 8, 26 and 52
Levodopa-equivalence dose	Levodopa-equivalence dose	0, 8, 26 and 52
Changes in medication	Changes in concomitant medication	0, 8, 26, 52 and 56–60
Side effects	Modified Toronto Side Effects Scale and reporting of other adverse events	0, 8, 26 and 52
Carer-reported outcomes:		
Carer burden	EQ-5D-5L and Quality of Life questionnaire for carers (QOL carers) will be completed if the participant has a carer	0, 8, 26 and 52
Number of dropouts	Number of participants who dropout from the trial	8, 26 and 52

Following the 52 week assessment, the trial medication will be tapered off as above, and the participants will be assessed at 56–60 weeks on the BDI-II. MDS-UPDRS, MoCA concomitant mediation and reporting of Adverse Events.

### Sample size

A total of 408 participants will be recruited in the full trial. The primary outcome is change in depressive symptoms measured using the BDI-II after 8 weeks of trial treatment. For 90% power and a significance level of 0.025 (for each comparison to preserve studywise alpha), 113 participants are needed per group to detect a 3-point BDI-II difference [SD for change 6.35] [20, 21] for the escitalopram–placebo and the nortriptyline–placebo comparisons at 8 weeks. Allowing for 20% attrition, 136 participants will be required per randomised group (408 overall).

With this sample size, the study will have 90% power (1-beta) to find a mean difference of change of 3 points on the Movement Disorders Society Unified Parkinson’s Disease Rating Scale (MDS-UPDRS) motor subscale (part 3), with a nominal alpha of 0.025 for each active comparison, taking the effective SD from [22].

For the estimation of adverse reactions we will use the Modified Toronto Side Effects Scale which elicits rates of side effects. We will use estimation and provide 95% confidence intervals around the difference in percentage side effects for each item, however the width of these confidence intervals will depend upon their position on the binomial distribution. Thus, a trial with 136 subjects in each experimental condition will provide 95% confidence intervals on the comparison between active agents ± 12% when the rate of events is around 50%, and ± 7% when the rate of events is around 10%.

### Statistical analysis

A detailed statistical analysis plan will be produced prior to analysis and agreed by the Trial Steering Committee (TSC). Descriptive analyses will examine the baseline characteristics of the treatment groups. The primary analysis will be the comparison of change in the BDI-II, conditional on baseline score, between the escitalopram and placebo group and between the nortriptyline and placebo group. As an exploratory analysis, we will provide an estimate of the difference in mean BDI-II score and 95% confidence intervals between the two active treatments. Secondary analysis will describe the number of participants experiencing adverse events on the Modified Toronto Side Effects Scale. A comparison between the escitalopram and nortriptyline arms for the following secondary outcomes will be made: motor examination (part III), the individual and combined part I and part II (motor and non-motor experiences), the motor complications part of the MDS-UPDRS (part IV) scores and their changes from baseline; of the clinical global impression (CGI) change in health score, the number of adverse events and of drop-outs; and all other secondary outcome measures. Secondary analyses will be performed for outcomes at 8 weeks and at 26 and 52 week and after withdrawal for those who have completed these assessments (blinded long term follow up with placebo group). Multivariate joint models will be used to explore the relationship between stopping treatment and the BDI-II.

Planned sensitivity analyses of different disease stages (Hoehn and Yahr stages), severity of depression (BDI-II categories of mild-moderate-severe), presence of anxiety and cognitive impairment will be performed. Further sensitivity analyses will be decided after the initial data analysis.

### Economic analysis

We will calculate the net monetary benefit (NMB) of (i) escitalopram plus standard psychological care, (ii) nortriptyline plus standard psychological care, and (iii) standard psychological care alone, to evaluate which of the three treatment options is the most cost-effective treatment of depression in PD. NMB will be calculated as the cost per quality adjusted life year (QALY) gained of each treatment option multiplied by a willingness to pay for a QALY gained. The primary analysis will be from a health and social care cost perspective and including participant data only, with secondary analyses including carer data. The analysis will use trial data only, and will report the NMB at 8 weeks, in line with the trial’s primary outcome measure, and at 52 weeks.

### Internal Pilot study

The trial will include an internal pilot study in the first 6 months with the aim to recruit 46 participants. The results of this pilot study will determine progression to the full trial. Aggregated data will be collected on eligibility, uptake/recruitment, reasons for declining to participate where possible, adherence and attrition, as well as completion of outcome measures. The main progression criterion will be the ability to recruit, with additional consideration of rate of clinically significant adverse reactions, loss to follow up before primary outcome and adherence to trial medication through pill count.

### Patient and public involvement

The trial design and all patient-facing documents received extensive input from our Parkinson’s patient group. There is ongoing input on trial management and engagement from the group, including advice on trial procedures, engagement and dissemination of study information.

### Data management

Data management will be complete by the UCL Comprehensive Clinical Trials Unit. Data will be entered onto the ADepT-PD electronic database and monitored by the UCL comprehensive clinical trials unit. Data with personal information will be held coded and in a pseudo-anonymised fashion on a university network which is password protected. Adverse Events data will be coded using the Common Terminology Criteria for Adverse Events version 5.0. Range checks and data formats are checked on the electronic database. The database software provides a number of features to help maintain data quality, including maintaining an audit trail.

### Trial oversight

The UCL Comprehensive Clinical Trials Unit as the sponsor has overall responsibility for the trial, including site and investigator selection. The trial is overseen by the Trial Steering Committee which consists of external consultees from clinical, scientific backgrounds and people with lived experience of PD. The Independent Data Monitoring Committee whose members provide expert knowledge/advice on different aspects notably clinical expertise on depression in Parkinson disease, conduct of clinical trials and statistical analysis of trial data, meet at scheduled time points throughout the duration of the trial to review interim trial data and safety data.

## Discussion

Depressive symptoms are a common and disabling feature of Parkinson’s disease from the early disease stages with increasing prevalence with advancing disease [23]. However, at present, there is insufficient evidence to guide the most appropriate treatment. Optimisation of antiparkinsonian treatment with dopaminergic medication may improve depressive symptoms, and psychological treatments should also always be considered, but these approaches are often not sufficient or feasible. Previous trials have suggested improvement with a number of different antidepressants, but trials have been small and with inconsistent results, and there are concerns about adverse effects, including deterioration of parkinsonism and cognitive worsening with antidepressants [11, 24, 25]. Trial evidence is most favourable for tricyclics, resulting in some guidelines suggesting their preferential use, but concerns about adverse effects in un-selected populations often limit their use in routine clinical care. A commissioned call by the HTA therefore sought to establish a placebo-controlled trial of a tricyclic antidepressant and an SSRI in patients with depressive symptoms in patients with PD in addition to routine clinical care.

This trial, which will compare nortriptyline as well as escitalopram against placebo in their effectiveness for depressive symptoms in patients with PD, is set in a routine NHS setting across the UK, and therefore has the potential to change clinical practice based on real-life trial evidence. It will examine the effect on depressive symptoms, anxiety, capability and health-related quality of life in these patients over an 8 week period, with 12 months continuation to examine longer term effectiveness. In addition, it will also establish the safety of these medications in this population, in particular with regard to change of parkinsonism and change in cognitive scores. This is also of relevance as nortriptyline has been reported in a several pre-clinical models to attenuate alpha-synuclein aggregation, which underlies PD pathology [15]. The current trial therefore also provides the opportunity to examine the potential of this compound to change progression of motor symptoms, using detailed motor assessments during On-periods, and Off-periods where possible, as well as wearable sensors. These outcomes will be examined over the follow up period of 12 months.

A range of non-motor symptoms of PD will be examined, and adverse events studied systematically with a side effects scale in addition to standardised reporting. Furthermore a health economic evaluation will be performed to assess whether clinical effectiveness, if found, will also be translated into cost effectiveness.

## Supplementary Information

**Additional file 1: Table 1.** Participant timeline.

## Data Availability

Not applicable. The trial results will be published in a peer reviewed journal and made available at the end of the trial. The Investigators will have access to the final trial dataset.

## Abbreviations

ADepT-PD: Antidepressant trial in Parkinson’s
BDI-II: Beck Depression Inventory-II
CGI: Clinical Global Impression
DSM V: Diagnostic and Statistical Manual of Mental Disorders, Fifth Edition
HTA: Health Technology Assessment
ICECAP-O: ICEpop CAPability measure for Older people
NMB: Net Monetary Benefit
MAO: Monoamine oxidase
MDS: Movement Disorder Society
MoCA: Montreal Cognitive Assessment
MDS-UPDRS: Movement Disorder Society Unified Parkinson’s Disease Rating Scale
NICE: National Institute for Health and Care Excellence
PD: Parkinson’s disease
QALY: Quality adjusted life year
REC: Research Ethics Committee
TCA: Tricyclic antidepressant
TSC: Trial Steering Committee
SSRI: Selective Serotonin Reuptake Inhibitor
