# Supplementary material for: Antidepressants Trial in Parkinson's Disease (ADepT-PD): protocol for a randomised placebo-controlled trial on the effectiveness of escitalopram and nortriptyline on depressive symptoms in Parkinson’s disease
Source: BMC Neurol. 2022 Dec 12;22:474. doi: 10.1186/s12883-022-02988-5 (PMC9743717; doi:10.1186/s12883-022-02988-5)
Supplement: Supplementary file 1 — Additional file 1: Table 1. Participant timeline. [file 12883_2022_2988_MOESM1_ESM.docx]

Supplement

Table 1 Participant Timeline

|  | **Screening/**  **Baseline Visit**  **(week 0)^a^** | | **IMP dispensing receipt call**  **(week 1) ^b^** | **Escalation period calls**  **(week 2, 4, 6)^c^** | **8 Week Visit^d^** | **IMP dispensing receipt call**  **(week 12)** | **26 Week Visit^d^** | **IMP dispensing receipt call**  **(week 26)** | **IMP dispensing receipt call**  **(week 39)** | **52 Week Visit^d^** | **Tapering period calls**  **(week 54, 56, 58)^e^** | **End of Study Visit**  **(weeks 56-60)^f^** |
| --- | --- | --- | --- | --- | --- | --- | --- | --- | --- | --- | --- | --- |
| **Flexibility of schedule ± days** | **0** | |  |  | **+/-1 week** |  | **+/-2 week** |  |  | **-1 week** |  | **+4 weeks** |
| **Visit number** | **1** | | 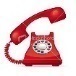 | 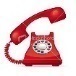 | **2** | 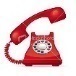 | **3** | 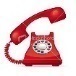 | 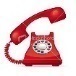 | **4** | 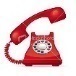 | **5** |
|  | **Screening** | **Baseline** |  |  |  |  |  |  |  |  |  |  |
| Informed consent | ● |  |  |  |  |  |  |  |  |  |  |  |
| Eligibility screen | ● |  |  |  |  |  |  |  |  |  |  |  |
| Depressive Symptoms Evaluation | ● |  |  |  |  |  |  |  |  |  |  |  |
| BDI-II | ● |  |  |  | ● |  | ● |  |  | ● |  | ● |
| MoCA | ● |  |  |  | ● |  | ● |  |  | ● |  | ● |
| Demographics | ● |  |  |  |  |  |  |  |  |  |  |  |
| Medical history | ● |  |  |  |  |  |  |  |  |  |  |  |
| Concomitant medications (levodopa-equivalence dose) | ● |  |  |  | ● |  | ● |  |  | ● |  | ● |
| Concomitant psychological therapies | ● |  |  |  | ● |  | ● |  |  | ● |  |  |
| Vital signs^g^ | ● |  |  |  | ● |  | ● |  |  | ● |  | ● |
| Pregnancy testing (if applicable) | ● |  |  |  | ● |  | ● |  |  | ● |  |  |
| Randomisation |  | ● |  |  |  |  |  |  |  |  |  |  |
| Trial medication/dosing diary discussion^h^ |  | ● |  |  | ● |  | ● |  |  | ● |  |  |
| Check receipt of trial medication |  |  | ● |  |  | ● |  | ● | ● |  |  |  |
| Adverse events |  |  |  | ● | ● |  | ● |  |  | ● |  | ● |
| MDS-UPDRS^i^ |  | ● |  |  | ● |  | ● |  |  | ● |  | ● |
| Timed Sit-Stand-Walk Assessment |  | ● |  |  | ● |  | ● |  |  | ● |  | ● |
| PHQ-9 |  | ● |  |  | ● |  | ● |  |  | ● |  |  |
| ICECAP-O |  | ● |  |  | ● |  | ● |  |  | ● |  |  |
| Parkinson’s anxiety scale |  | ● |  |  | ● |  | ● |  |  | ● |  |  |
| CGI (change in health) |  |  |  |  | ● |  | ● |  |  | ● |  |  |
| EQ-5D-5L |  | ● |  |  | ● |  | ● |  |  | ● |  |  |
| Modified Toronto Side Effects Scale |  | ● |  |  | ● |  | ● |  |  | ● |  |  |
| Modified CSRI (incorporating modified iVICQ) |  | ● |  |  | ● |  | ● |  |  | ● |  |  |
| QOL-carer^j^ |  | ● |  |  | ● |  | ● |  |  | ● |  |  |
| EQ-5D-5L carer^j^ |  | ● |  |  | ● |  | ● |  |  | ● |  |  |
| Pill count (IMP Review & Compliance) |  |  |  |  | ● |  | ● |  |  | ● |  | ● |
| Dose escalation / reduction reminders^k^ |  |  |  | ● |  |  |  |  |  |  | ● |  |
| Movement Sensor (optional) ^m^ |  | ● |  |  |  |  | ● |  |  |  |  | ● |

^a^ The Screening and Baseline assessments can occur on the same day, or be completed within 14 days

^b^ Week 1 will commence when the participant takes their first dose of IMP

^c^The Escalation period calls will occur at the end of Week 2, Week 4 and Week 6 for participants aged 65 and under, and at the end of Week 2 for participants aged over 65 (or with hepatic impairment).

^d^Where attendance at the trial site is not possible, efforts will be made to collect the information remotely (e.g. via telephone).

^e^ The Tapering period calls will occur at the end Week 54, Week 56 and Week 58 for participants aged 65 and under, and at the end Week 54 for participants aged over 65 (or with hepatic impairment).

^f^ The End of Study visit will take place at Week 60 for participants aged 65 and under and at Week 56 for participants aged over 65 (or with hepatic impairment).

^g^ If the patient is seen remotely, the vital signs do not need to be performed

^h^A member of the site trial team will provide the participant with copies of the Dosing Diaries which confirms how much trial medication the participant has to take daily and is also used for the participant to record the actual number of tablets they take each day.

^i^ If the participant has Off-periods, at baseline and one-year follow-up, it should be attempted to also assess the participants during a practically defined Off-period e.g. before the first dose of antiparkinsonian medication, with an additional assessment of the Movement Disorder Society – Unified Parkinson’s Disease Rating Scale (MDS-UPDRS) motor assessments. This Off-period assessment could be done during an assessment of the participant at home.

jOnly if participant has a carer.

^k^A member of the local site research team will contact the participant at the relevant times during the relevant escalation/tapering periods (i.e. every 2 weeks when the dose is being escalated at the start of the participant’s trial treatment and every 2 weeks when the dose is being reduced at the end of the participant’s trial treatment) to remind the participant when the dosage needs to be changed.

^l^ Worn for 7 days, before being returned in the post in a provided stamped addressed envelope
